# Marine NMEA 2000 Smart Sensors for Ship Batteries Supervision and Predictive Fault Diagnosis

**DOI:** 10.3390/s19204480

**Published:** 2019-10-16

**Authors:** Emilio García, Eduardo Quiles, Antonio Correcher, Francisco Morant

**Affiliations:** Instituto de Automática e Informática Industrial, Universitat Politècnica de València, 46022 Valencia, Spain; egarciam@isa.upv.es (E.G.); ancorsal@ai2.upv.es (A.C.); fmorant@isa.upv.es (F.M.)

**Keywords:** marine sensor system, NMEA 2000 network, ship networking technology, batteries, predictive fault diagnosis

## Abstract

In this paper, an application for the management, supervision and failure forecast of a ship’s energy storage system is developed through a National Marine Electronics Association (NMEA) 2000 smart sensor network. Here, the NMEA 2000 network sensor devices for the measurement and supervision of the parameters inherent to energy storage and energy supply are reviewed. The importance of energy storage systems in ships, the causes and models of battery aging, types of failures, and predictive diagnosis techniques for valve-regulated lead-acid (VRLA) batteries used for assisted and safe navigation are discussed. In ships, battery banks are installed in chambers that normally do not have temperature regulation and therefore are significantly conditioned by the outside temperature. A specific method based on the analysis of the time-series data of random and seasonal factors is proposed for the comparative trend analyses of both the battery internal temperature and the battery installation chamber temperature. The objective is to apply predictive fault diagnosis to detect any undesirable increase in battery temperature using prior indicators of heat dissipation process failure—to avoid the development of the most frequent and dangerous failure modes of VRLA batteries such as dry out and thermal runaway. It is concluded that these failure modes can be conveniently diagnosed by easily recognized patterns, obtained by performing comparative trend analyses to the variables measured onboard by NMEA sensors.

## 1. Introduction

Vessels are essentially floating complex systems, such as freighter ships, carrier ships, cruise ships, ship factories, oceanographic research ships, and battle ships, etc. All of these vessels are equipped with navigation, propulsion, power generation, distribution, and other systems of life support. For the control and supervision of such systems, the existence of common standards of communication networks would be desirable. As in other distributed control schemes, the transfer of data in ships is done with networks of low-level control, of real-time features, and of critical natures, with temporary response restrictions. They include less restrictive hierarchical networks not directly related to the safety of the ship, up to supervision, planning, and business management systems.

Decision-making that affects the operation of the ship, involving safety, crew, passengers, cargo and the environment, is generally performed on the bridge or other control centers. To make decisions efficiently and carry them out requires access to many of the information systems on board. Systems of special relevance can include navigation, weather forecasting, power generation and storage, engines and machinery, data processing, messages and alarms, etc. The special risks involved in an aggressive environment such as marine navigation require adequate levels of security, availability, redundancy, and latency of communications networks. Although local office-type networks can work at the same speed, from the point of view of reliability they are unadvisable.

A large number of sensors, controllers, electronic devices and systems must be installed on shipboard, and they generate a huge amount of data and information. Such information must be exchanged reliably to aid system integration and safe navigation [1,2]. These devices necessarily feed from power sources for their adequate operation. One of the most critical scenarios to face is the temporary or permanent loss of power supply, which can lead to placing the ship and crew at risk. In this context, redundant supply structures and predictive fault diagnosis techniques are concepts to consider. The permanent supervision and trend analysis of the most critical elements can allow for the detection of anticipatory symptoms—with adequate reaction margins—before reaching a point of permanent failure.

Specifically, issues concerning power generation, status supervision, alarms, prognosis, and the control of devices such as generators, alternators, hybrid inverters, and shore-power, are tasks to be considered. In addition, electrical distribution tasks such as the delivery of power on vessels, identification of loads, load sharing, and virtual breakers are important issues.

Ship navigation involves different critical operations where no uncertainty in decision making is acceptable. Such uncertainty can subject crew, passengers, cargo, or the ship itself to conditions of potential danger. A diagnosis of predictive failures is essential for a safe navigation. This diagnosis is based on the permanent analysis of the health condition of the system, through the detection and analysis of different symptoms that precede system’s failures. This analysis can be done using time-series data of certain system parameters. These techniques for predictive diagnosis allow for generally adequate margins or reaction capacities in the application of predictive maintenance operations.

In order to maintain a safe navigation, ships require that many systems are continuously available, i.e., navigation, communication, and weather forecast equipment. If a failure of the storage and power supply system affects the maneuvering system during a critical operation, the result can be a serious accident [3]. Therefore, the energy supply system from the batteries must have a monitoring availability [1]. Ships and aircrafts install redundant systems, especially for the most critical components, so that no single fault can result in a general failure.

In ships, battery banks are installed in chambers that normally do not have temperature regulation and that are therefore significantly conditioned by the outside temperature. In this paper, a specific method based on the analysis of the time-series data of random and seasonal factors is proposed for the comparative trend analyses of the internal temperature of batteries and the battery installation chamber temperature. The objective is to apply a predictive fault diagnosis to detect any undesirable increase in battery temperature due to a prior heat dissipation process failure, to avoid the development of the most frequent and dangerous failure modes of VRLA batteries—such as dry out and thermal runaway. It is concluded that these failure modes can be conveniently diagnosed by easily recognized patterns obtained by performing comparative trend analyses to the variables measured onboard by National Marine Electronics Association (NMEA) sensors.

The advantage of the diagnostic method based on the comparative trend analyses proposed in this work is that it focuses on the assignable cause that precedes the dry out and thermal runaway failures, which is the loss of the operating capacity of the heat dissipation process of the battery banks. The heat dissipation process of battery banks in real navigation conditions does not have a specific nature. It depends on the characteristics of the installation chambers (which do not remain invariable in time), the materials used, the size of the heat dissipation areas, and the ventilation conditions, etc. All these factors affect the batteries in a variable way, depending on the type of batteries and their intrinsic aging conditions.

In this study the operation and tests of the batteries have been made in real navigation conditions using a NMEA 2000 sensor network. As stated before, the possibility of using refrigerated chambers for battery installation is not feasible in most cases, nor from the economic point of view or from the energy consumption point of view.

In Section 2 of this paper existing standards of communication in the marine environment are reviewed. Section 3 reviews the causes and models of battery aging, including the types of failures and predictive diagnosis techniques for batteries used for assisted and safe navigation. In Section 4, the NMEA 2000 network is partially described, and especially the part dedicated to the sensor devices for the measurement and supervision of the parameters inherent to the energy storage and supply. In Section 5, the proposed method for onboard battery supervision and predictive fault diagnosis is presented. In Section 6, NMEA 2000 sensors and devices measurements, trends and registers, of the main battery parameters and their interpretation are presented. Finally, in Section 7 some conclusions about the advantages of the proposed method for fault identification by comparative trend analyses are drawn.

## 2. Brief Review of Marine Network Communication Technologies and Standards

Before the year 2000, some attempts of standardizing communication networks in ships were made. Various manufacturers developed different systems, however, differences in requirements made it difficult to find one common standard for the networks used in the different parts of the ship. IEC 61162-1 defines about 50 talker identifiers for individual types of navigation and radio communication equipment alone. In [4] approximately 130 different functions on were identified on board the ship, with most of them associated with some type of computerized equipment that performs supervision and control. This fact justifies that a data network standard must be able to support many different systems and support easy interconnection.

With regard to maritime navigation, a new standard for linear network communication—NMEA 2000—was presented in 2001 by the National Marine Electronics Association (NMEA) [5,6]. It was based on a Controller Area Network (CAN) network, as a solution to the growing expectations dealing with data exchange among electronic ship devices. Previously, in March of 1983, NMEA 0183 was introduced as a voluntary industry standard, which used a simple ASCII serial communications protocol [7,8].

Some features of the data connection layer of the NMEA 2000, like in the physical layer, are determined by the choice of CAN as the main network. The NMEA 2000 fully makes use of the international standard of the ISO 11783-3 data connection layer, which is virtually identical to the SAE J1939-21 standard (i.e., the specification of the CAN for lorries, delivery trucks and trailers). Additional requirements contained in the NMEA 2000 ensured better copying, with special types of data and formats transmitted through a navigational device and supported by the special construction of such devices [9].

In 2007 CANopen as IEEE P1551.6 was proposed as belonging to the IEEE 1451 family of Smart Transducer Interface Standards for sensors and actuators [10,11]. 

Concurrently, taking in mind mainly big ships, the Working Group 6 (WG6) of Technical Committee 80 (TC80) of the IEC defined a set of IEC 61162 standards for “Digital interfaces for navigational equipment within a ship”, divided into four parts [12]:

- Part 1: IEC 61162-1 single talker and multiple listeners (also known as NMEA 183).
- Part 2: IEC 61162-2 single talker and multiple listeners, high-speed transmission.
- Part 3: IEC 61162-3 serial data instrument network (also known as NMEA 2000).
- Part 450: IEC 61162-450 multiple talkers and multiple listeners–Ethernet interconnection (also known as Lightweight Ethernet).

Subgroup TC80/WG6 specified the use of Ethernet for on-board navigation networks. The specification was limited to the transport of NMEA sentences subject to the definition made in 61162-1 on IPv4. Due to the low amount of complexity of the protocol, it was called Lightweight Ethernet (LWE), and was presented at the ISIS 2011 symposium [13].

IEC 61162-460: 2015 (E) is a complement to the IEC 61162-450 standard, seeking the introduction of higher security standards, and improving network integrity. The first edition was published in 08/2015.

Some of the first ship data network standards published were the US Navy’s SAFENET (Survivable Adaptable Fiber optic Embedded Network) standards I and II [14] in 1988, and ATOMOS [15] from 1992 to 2001, developed through four European research projects. Both used token passing on the data-link layer to get better real time performance and more deterministic latency from the network. Meanwhile, due to constant advances in higher-level protocols, Internet Protocols (IP) became predominant with higher speeds and advances, with Ethernet switches making the latency argument less relevant. Since 1990, Ethernet began to be considered as a more deterministic network, and, therefore more used.

In the period 1991–1993 a Norwegian research project, MITS (Maritime Information Technology Standard) was developed and subsequently, between 1993–1996 the MITS protocol was implemented on several ships, based on a single non-redundant Ethernet on the physical layer and TCP/IP protocols up to the transport layer [16]; however, mainly due to its lack of standard redundancy support its uptake was delayed. 

Standard redundancy support and fully redundant network system based on dual Ethernets and the IP protocols were specified in the project PISCIS (1998–2000), taken up and developed by the IEC 61162-400 series of standards [15].

The new work item 80/506/NP on an Ethernet based interface standard was proposed in 2007 by Sweden to the IEC and accepted in March 2008. The IEC TC80/WG6 went to work on its development and the final version was published in April 2011 as IEC 61162-450 [17]. 

This TC80/WG6 subgroup has specified with IEC 61162-450 and IEC 61162-460 standards the use of Ethernet and safety security conditions for shipboard navigational networks, taking into account the new developments in legislation that make it necessary to look closer at improved system integration tools such as data networks [18,19].

Other contributions in the context of IT standards were made through major integration projects such as Flagship, especially in the development of IEC 61162-450 and the final stages of ISO 28005 [4].

Currently, there are projects such as Signal K, which has been proposed as a solution of the next generation for the exchange of marine data. It not only allows communication between instruments and sensors aboard a single ship, but also aims to share data among several ships, navigation aids, bridges, marinas and other land resources. It is designed to be easily used by web and mobile applications and to connect modern ships to the concept of the Internet of Things. It uses a “Smart” Gateway that converts existing NMEA data into Signal K, and its installation consists simply of wiring the gateway to NMEA0183 and/or NMEA 2000 networks and plugging the gateway into a wireless router [20].

## 3. Supervision and Predictive Fault Diagnosis of Batteries

If properly designed, built, and maintained, a battery can provide many years of reliable service. A new battery might not initially provide 100% capacity. The capacity typically improves over the first few years of service, reaches a peak, and declines until the battery reaches its life limit. A reduction to 80% of the rated capacity is usually defined as the end of life for a lead-acid battery. Below 80%, the rate of battery deterioration accelerates, and it is more prone to sudden failure resulting from a mechanical shock or a high discharge rate. Note that even under ideal conditions, a battery is expected to eventually wear out [21]. 

With the objective of maximum availability of the energy storage system, the storage capacity and the useful life of the batteries depends to a large extent on a suitable management of their use [22]. Overloading excessively as well as under-charging or over-charging a battery can deteriorate it. Condition monitoring devices can adjust the use of batteries to extend their life. In addition, they can estimate the amount of energy stored in the batteries to plan power usage and charging cycles, and they can control the condition of batteries allowing to apply predictive maintenance techniques to determine when to replace them [23,24].

The dynamic behavior of battery operation has been studied by a large number of researchers [25]. The models developed years ago with lead-acid batteries [26,27,28] keep some similarities with nickel-cadmium batteries, and lithium batteries. Nickel-cadmium (NiCd) chemistry is similar in some respects to lead-acid in that there are two dissimilar metals in an electrolyte. However, in NiCd batteries the potassium hydroxide (KOH) does not enter the reaction like sulfuric acid does in lead-acid batteries. The making is similar to lead-acid in that there are alternating positive and negative plates submerged in an electrolyte [29]. A review of mathematical models of both lithium and NiCd batteries, developed at the University of South Carolina, is presented in [30]. The dynamic models of lithium batteries have been extensively developed by many authors. These range from simple models with a resistance (R) or a capacitor-resistance (RC) in parallel [31,32,33], to more complex models where phase change elements and coils are introduced [34,35]. The researchers modus operandi was mostly the same: Placing these elements in series and incorporating particularities to achieve higher levels of adjustment in the electrical behavior of the battery [36,37].

In addition to these electrical models, other mathematical models have been developed to estimate the parametric variations depending on the values associated with the time of use/disuse and variations of the temperature of the battery [38]. These models are completed by adding the effects of aging to determine the rest of useful life (RUL) of the battery. The main factors that affect accelerate battery aging mechanisms are: Temperature (T), depth of discharge (DOD), state of charge (SOC), and discharge velocity (C-rate) [39,40,41]. 

To monitor the useful life of batteries, the aging phenomenon must be analyzed. Batteries age both when they are stationary (calendar ageing) and when they are subjected to a cyclic operation (cycling ageing) [25]. In the first case, apart from the passage of time itself, there are mainly two factors involved in aging: The temperature and the SOC. The temperature affects according to an exponential relation that is explained by the Arrhenius equation [42], while the SOC does so linearly [43,44]. On the other hand, in aging due to cycling, the DOD and the C-rate also intervene [45]. The first one does it by means of a logarithmic relation whereas the C-rate does it by means of a second-degree polynomial [46,47]. 

In practical terms, these effects translate into two repercussions: An increase in the internal resistance of the battery and a loss of capacity [48]. Although there are also effects on the RC elements, they only act on the instantaneous response of the battery to sudden changes in the current. For practical purposes, a correlation can be established between the loss of capacity of the battery and its aging which, although it happens imperceptibly, will occur constantly. 

The most visible consequence of the aging of a battery is the gradual loss of its capacity, which is established through the parameter of the state of health of the battery (SOH). The SOH is calculated as the quotient between the current capacity (Cap) of the battery and the initial capacity (Cap_ini_) according to Equation (1).

SOH = Cap/Cap_ini_ (1)

The SOH will serve to determine the limit of its operation, that is, it is the parameter that defines the end of life of a battery in each application. In the literature on this subject, there are experimental tests carried out in laboratories where programmed tests have been performed under specific conditions and patterns to analyze the aging of batteries and other related parameters [49,50,51,52,53].

In many cases, when considering the actual operating conditions of batteries, the determination or estimation of aging parameters through the measurement of their internal variables may be inaccessible to sensors. Measurements can be very difficult, expensive, or intractable, and therefore the mathematical models obtained in the experimental laboratory conditions do not conveniently adapt to the random conditions of complex degradation associated with the actual operating regime to which batteries are subjected in maritime navigation conditions. In such conditions the gradual degradation of the batteries occurs due to cumulative effects of multi-parametric character where the degree of contribution of each of them is very difficult to quantify. Under real operating conditions, on-line techniques of trend analysis to monitor the true SOH of batteries are more appropriate [54].

One of the important aspects of diagnosis is to determine the end of the useful life of the batteries with the possibility of proceeding to an adequate maintenance in time and form. However, it is also necessary to consider risks associated with certain typical failures of catastrophic nature, whose consequences can be manifested in the short term. These types of failures are named and defined in the next section.

### Failure Modes of VRLA Lead-Acid Batteries

It is known that batteries have different failure modes depending on their technology [55]. Nowadays, one of the most used type of batteries in maritime navigation are those of the VRLA type, due to their comparative benefits and relative low cost in both installation and maintenance, energy density, and safety. For these kind of batteries, the associated characteristic failure modes are those described below [56,57].

- Dry out (loss-of-compression)
- Plate sulfation
- Soft and hard shorts
- Post leakage
- Thermal runaway
- Positive-grid corrosion

Some of the failure modes listed above, especially dry out, positive-grid corrosion and thermal runaway, are strongly dependent on the increase in the internal battery temperature T_in_, which in turn depends under normal conditions largely, but not exclusively, on the external temperature T_ex_ due to weather conditions. The outside temperature, T_ex_, is “filtered” by the battery installation chamber and becomes the ambient temperature T_amb_.

T_in_ has a strong influence on aging, grid-corrosion rates, and rates of water loss (dry out) due to evaporation or hydrogen evolution at the negative plates (self-discharge), which all increase with increasing temperature. On the other hand, a (moderate) temperature increase may improve service life in applications involving severe cycling [58]. 

Dry out is a phenomenon that occurs and is accelerated due to excessive heat (lack of proper ventilation or in other words, heat accumulation inside the battery due to a prior failure from the heat dissipation process) and over-charging, which can cause elevated internal temperatures, high ambient (installation chamber) temperatures, and contributes decisively to grid corrosion. Up to 82–85% of the failures exhibit signs of dry out [57]. It is often a secondary result of some failure modes and special inducer of others, such as thermal runaway. Under normal operating conditions the typical failure mode for a VRLA battery is negative-strap corrosion, whereby the loss of electrolyte will be gradual. At elevated internal temperatures, the sealed cells will vent through the pressure relief valve (PRV). When sufficient electrolyte is vented, the glass matte is no longer in contact with the plates, thus increasing the internal impedance and reducing battery capacity. 

In turn, the causes of the increase in the internal temperature of the battery are the ambient temperature T_amb_, the V_float_ current, and the ripple current effect. 

Thermal runaway is a condition in which the battery temperature increases rapidly resulting in extreme overheating of the battery, therefore the battery can melt, catch on fire, or even explode. Thermal runaway can only occur if the battery is at a high-ambient temperature and/or the charging voltage is set too high [59,60]. Although a runaway failure is less frequent, it can have serious consequences of a critical nature. Thermal runaway occurs when a battery’s internal components melt-down in a self-sustained reaction. As the battery accepts current, its internal temperature rises. The rise in temperature reduces the battery impedance, causing it to accept more current from the charger. The higher current further heats the battery. Thermal runaway begins when the heat produced by the reaction, that increases exponentially, exceeds the heat removed, that increases linearly. The surplus heat raises the temperature of the reaction mass, which causes the rate of reaction to increase. This in turn accelerates the rate of heat production. An approximate rule of thumb suggests that reaction rate—and hence the rate of heat generation—doubles with every 10 °C rise in temperature causing the battery temperature to “runaway”. An upper limit of 126 °C [57] will eventually be reached when the electrolyte starts to boil, but once the electrolyte has boiled away, the temperature can climb even further to the point of plastic meltdown and possible fire [56].

Different batteries can be affected by the float voltage and the ambient temperature T_amb_ in different ways. Among diverse batteries of different make and model, there are significant differences in the float voltages that can cause different aging periods. The following factors contribute to the response variation in each battery behavior: Chemistry of the battery and construction, age of the battery, and especially chamber conditions where batteries are installed [61].

If the process of heat generation produced internally in the battery reaches an advanced uncontrolled phase, violent boiling will occur together with a rapid generation of gas causing in turn over-pressurization, which if not detected in time can cause catastrophic damage due to emissions of hydrogen, oxygen, hydrogen sulfide gas (an irritant), and atomized electrolyte. This process can cause a fire or explosion in the installation chamber [62]. In this scenario, the crew can be dangerously subjected to emissions of particles, liquids, and hot and toxic gases, with the risk of serious accidents.

Ripple current is another contributor for battery inner temperature increase. Battery manufacturers recommend that under normal float-charge conditions, battery ripple RMS (root mean square) voltage must be limited to <0.5% of the Direct Current (DC) voltage applied to the battery. This ensures that the instantaneous cell voltage will not fall below the open cell voltage or rise above the maximum float-charge voltage. It also eliminates the consequential battery heating that would occur from constantly cycling the battery through discharging and recharging states. Many early laboratory and real-world studies of lead acid (Pb) have shown that Alternating Current (AC) ripple may cause the cell to experience shallow discharge cycles, that in turn may lead to gassing, grid corrosion and internal heat generation [63,64].

It is well known that high temperature is the “killer” of all batteries, and that its effect varies depending on the manufacturer and model or the type of technology used in its manufacture. Lead acid at 95 °F (35 °C) will experience a 50% shortened life, while Ni-Cd will have a 16%–18% shortening of life [56,62]. For every 18 °F (10 °C) increase in battery temperature, battery life is halved. The increased temperature causes faster positive-grid corrosion as well as other failure modes. By holding a lead-acid battery at a temperature of 95 °F (35 °C) instead of the designed 77 °F (25 °C), a 20-year battery will last only ten years, a ten-year battery only five years, and so on. Increase the temperature by another 18 °F to 113 °F (45 °C), and a 20-year battery will last only five years. Therefore, in predictive trend analysis, the most important parameter to consider is the internal temperature of the batteries [56]. 

At the other end, the low-temperature range slows down the internal chemical reactions in any battery. The degree of reduced performances vary according to the technology also. For example, at temperatures around freezing, a VRLA may need capacity compensation of 20%. The lead-calcium cell using 1.215 specific gravity acid will require a doubling of capacity, while the Ni-Cd will need about an 18% increased capacity. 

Under ideal conditions, the trend analysis of certain battery parameters, especially temperature, impedance, capacity, and SOH, would be an excellent tool to observe how the batteries degrade over time, and when a decision can be made to replace them. However, as mentioned above, easy access to all of these parameters is not always available [65,66,67].

## 4. NMEA 2000 Network for Onboard Supervision and Fault Diagnosis

In the data connection layer of the NMEA 2000 network, the main functions of the CAN interface are: Generating the linear stream of bits, controlling the access to the network, as well as the controlling of errors and automatic transmission of error messages.

The CAN network is a linear, bit-orientated main. To avoid collisions and errors of transmission, the CSMA/CA method of the access to the main is applied, which determines that the reaction time of all drivers must be less than the transfer time of a bit [68].

NMEA 2000 consequently inherited these limitations that initially conditioned its use in large vessels. Some of these limitations were the length of the main speed versus transmission speed; since all the CSMA/CA devices have to work with the same speed and in long lines, differences of a signal due to delays, may appear. Currently, this restriction can be solved by using NMEA 2000 network segments with devices such as network bus extenders (NBE) [69].

NBE100 devices were developed to be able to extend the maximum node count up to 250, as well as the limit network trunk length and the cumulative drop length of a NMEA 2000 network. Without extenders a single network has a maximum of only nodes allowed, a network trunk length of 200 m, and a maximum cumulative drop length of 78 m.

In 2001, NMEA published its NMEA 2000 standard and was also adopted as an IEC 61162-3 standard. More than 140 renowned companies belong to the manufacturer’s list of NMEA 2000 developer members. NMEA 2000 has had a great penetration de facto in medium and small length vessels. Later in 2012, NMEA announced the project OneNet [70] to develop a common infrastructure to transport NMEA 2000 messages over Ethernet establishing standard gateway rules and supporting high-bandwidth applications such as video data transport, which is not possible using the NMEA 2000 network. OneNet does not replace NMEA 2000 using the physical and network layer standard based on the IEEE 802.3 Ethernet Standard, but complements the NMEA 2000 Standard and preserves existing and future NMEA 2000 messages (PGNs). OneNet should provide greater bandwidth, with up to 1 gigabit or faster transfer speed directly to the OneNet devices (400 times the speed of the NMEA 2000 CAN bus). It also provides greater scalability, as OneNet backbones may exceed 100 Mbps using other standard Ethernet physical layers such as Gigabit Ethernet and fiber optics supporting up to 65,024 physical devices, versus CAN bus 50 devices, allowing the creation of larger and more complex networks. With Power Over Ethernet (PoE) it allows each physical device to be separately powered by up to 15.4 watts directly from the Ethernet switch. OneNet is not recommended for real-time critical data, because the NMEA 2000 Controller Area Network (CAN) enables prioritization and guarantees that the message transmitted will always get through to certified devices. IEEE 802.3 cannot provide the same guarantee of message delivery.

**Figure 1:**
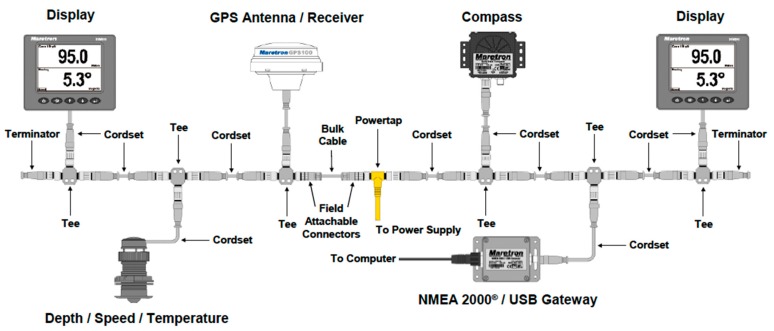
NMEA 2000 network [71].

**Figure 2:**
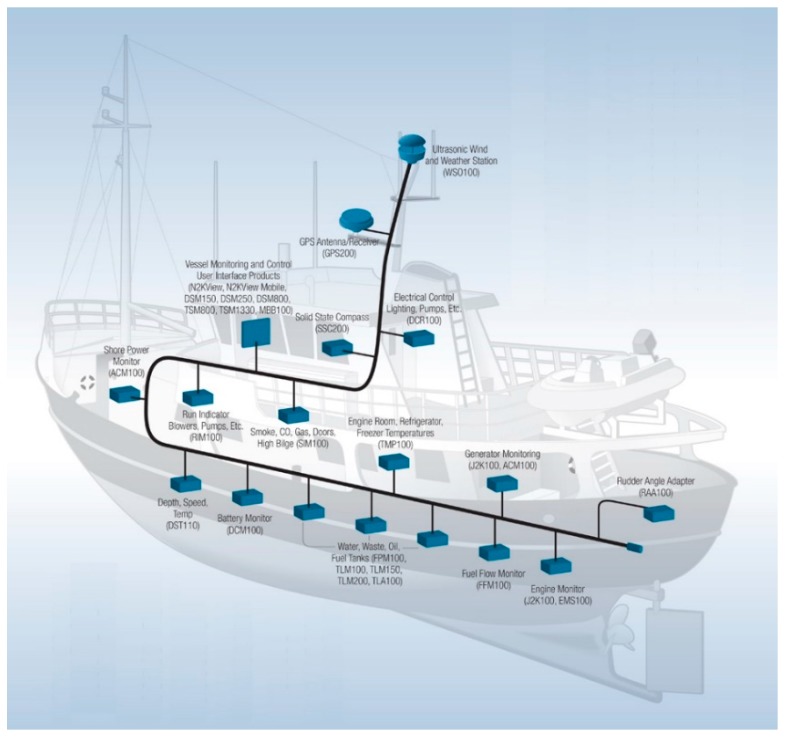
NMEA 2000 network vessel distribution [71].

The structure of NMEA 2000 is shown in Figure 1. A typical NMEA 2000 vessel distribution is shown in Figure 2.

In the NMEA 2000 network, devices are available for the measurement, monitoring, supervision and data processing of parameters from the navigation system, as well as graphical displays for the analysis of short-term trends. There are also devices for the registration of such data, such as the Voyage Data Recorder (VDR). These devices are reviewed in the following sections. 

### 4.1. Module and Sensor DCM100 Monitor Specifications

**Figure 3:**
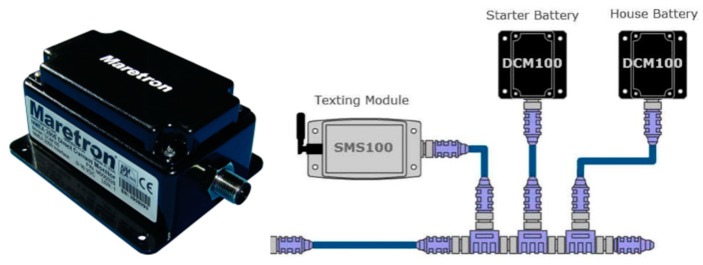
DCM100 direct current/battery module monitor [71].

**Figure 4:**
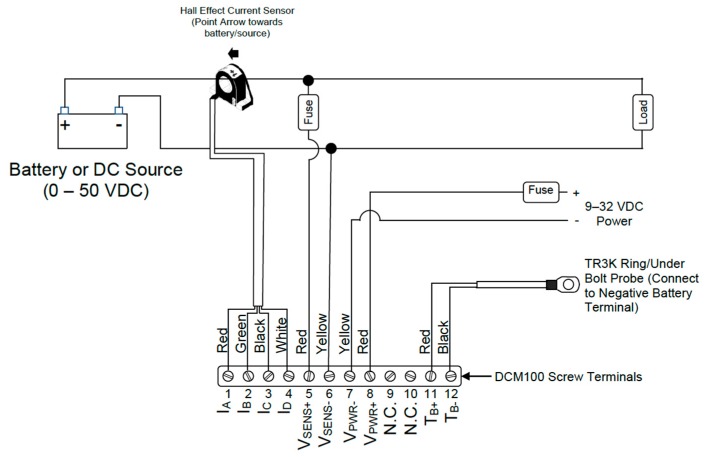
DCM100 connection diagram [71].

Maretron’s DCM100 (Figure 3) is a sensor device designed to operate within the harsh demands of the marine environment. It is able to monitor, by proper configuration, different DC power sources, such as batteries, alternators, convertors, solar cells, and both wind and marine turbines, transmitting data over the NMEA 2000 network. Figure 4 shows the hall effect current sensor and the DCM100 monitor module connection diagram.

If “Battery” type is selected, many battery-related options become available [72], where different types of batteries can be selected such as “Flooded/Wet”, “Gel”, “AGM”, and “Other”. A wide range of battery parameters can be selected and monitored:

- Battery Voltage
- Battery Current
- Ripple Voltage
- Battery Case Temperature
- State of Charge
- Time Remaining
- Battery Capacity
- Battery Types
- Charging Inefficiencies
- Charge Efficiency Factor (CEF)
- Discharging Inefficiencies
- Peukert Exponent
- Charge Efficiency Factor Calculation

### 4.2. Temperature Sensors

**Figure 5:**
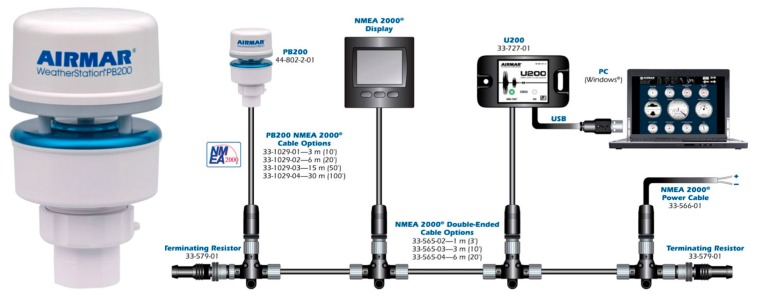
AIRMAR PB200 Weather Station.

The PB200 Weather-Station instrument is designed to output time series of data of weather parameters instantaneous values. Additionally, it is equipped with temperature and barometric pressure sensors that help to make trend analyses and forecasts on changing weather patterns (Figure 5). This weather-station is very complete and combined with the internal heading sensor, most of navigation parameters are provided if needed [73]. In this work, the integrated temperature sensor PB200 was used for the outside temperature time-series measurements [74].

**Figure 6:**
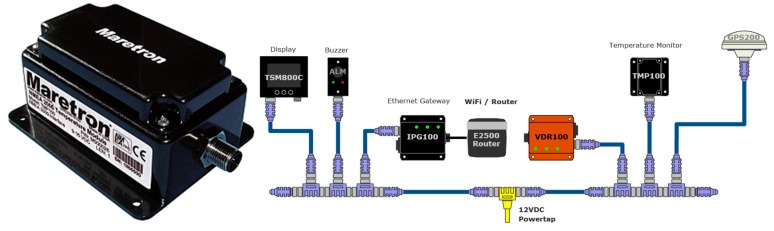
TMP100 Temperature Module [71].

**Figure 7:**
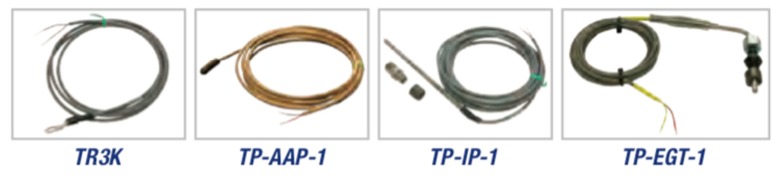
Temperature probes to be used with TMP100 temperature module [71].

Maretron’s TMP100 module measures the temperature for up to 6 temperature probes and reports the information over an NMEA 2000 network (Figure 6). The TMP100 supports up to four thermistor probes and two high-temperature thermocouple probes (Figure 7). Optional thermistor probes (−20 °C to 80 °C, or −4 °F to 176 °F) cover a wide range of applications including cabin air temperature, engine room air temperature, refrigerator/freezer temperature, under bolt temperature (inverters, charges, pumps, motors, etc.), tank temperatures (live well bait, hot water, etc.), and air duct temperatures. In these tests, the TP-AAP-1 TMP100 ambient air temperature probe (−20 °C to 80 °C or −4 °F to 176 °F) has been used for batteries installation chamber temperature measurements. A TR3K-Ring terminal probe (−20 °C to 80 °C or −4 °F to 176 °F), connected to the negative battery terminal has been used by DCM100 module to monitor inner battery temperature T_in_ [75].

### 4.3. NMEA 2000 Data Logging Capabilities

**Figure 8:**
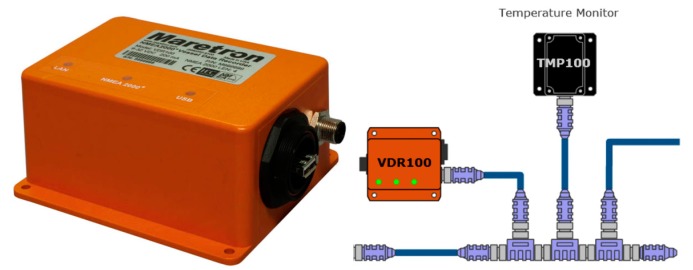
VDR100 Vessel Data Recorder [71].

The Vessel Data Recorder VDR100 (Figure 8) is a kind of “black box” that records all types of data, which circulate through the NMEA 2000 network associated with the navigation activity. The VDR works in solidarity with an associated N2KExtractor software [76], which is capable of recovering and monitoring historical data from a flash memory connected to the VDR100, and which can additionally select and export parameters in comma delimited files (CSV) for statistical and trend analysis purposes. The VDR device is equipped with three ports, one for connection to the NMEA 2000 network, another for connecting a flash memory, and the third for connecting to an Ethernet LAN. The VDR has been designed to support recording periods longer than one year [77].

**Figure 9:**
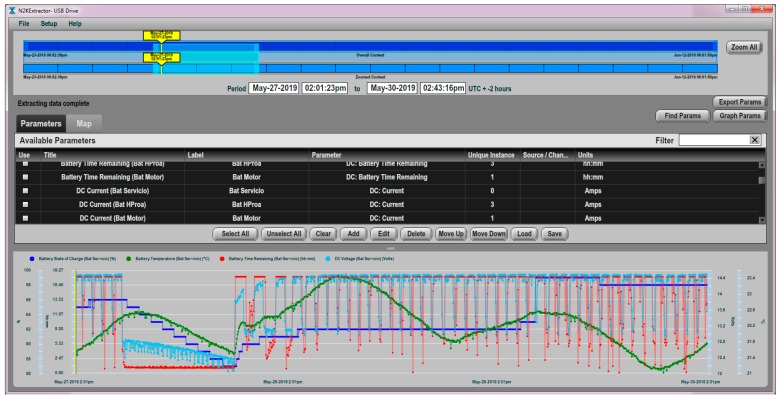
N2KExtractor workplace screen.

Additionally, this activity can be shown in the form of the time series of data of all the parameters that the DCM100 is able to monitor. In turn, the NMEA 2000 network has the ability to display on-line mobile windows of up to 1-week amplitude, and integrates the ability to perform treatment of alarms and emergencies. The N2KView software [78] or a Maretron DSM150/DSM250 display [79] can be used (Figure 9) to carry out the complementary activities of monitoring, treatment of alarms, messages and analysis of short-term trends. 

**Figure 10:**
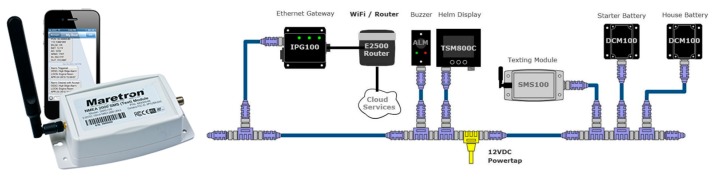
SMS100 short message service (Text) module [71].

Maretron’s SMS100 short message service (Text) module is a mobile or cellular modem dedicated to sending alerts or alarm messages to selected phone numbers (Figure 10). This device works jointly with sensors (specially DCM100) and software of the NMEA 2000 network. It provides cellular network coverage for warnings about battery failures and other alarms including the vessel’s position, bilge status, shore power voltage, wind speed, inside and outside temperature.

## 5. Fault Diagnosis Method

Trend analysis is a quantitative technique that can be used to identify potentially hazardous conditions based on past empirical data obtained from time series of data. A trend analysis can reveal a movement toward unacceptable, undesirable, or dangerous reliability, safety, or levels of assurance. Its application dramatically decreases uncertainty and emergency replacements allowing to make adequate decisions for maintenance planning [54]. In addition, if the particular trend model has a significant quantitative fit (for example, linear, quadratic, exponential), future predictions can be made [49,80,81,82].

In this work, a scenario under real weather conditions of navigation has been considered. This scenario is one in which a set of batteries are affected in terms of the dynamics of the temperature variable, by a set of numerical values associated with a time series of a stochastic process of seasonal character. 

One way of describing time series is based on the idea of decomposing the variation of a series into several basic components, which becomes especially interesting when in the series a certain tendency or certain periodicity is observed [58]. This descriptive approach consists of finding components that correspond to a long-term trend, a seasonal behavior and a random part. Of the three components reviewed, the first two are deterministic components, while the last one is random. Thus, the model of a time series can be denoted as:

X_t_ = T_t_ + S_t_ + I_t_ (2)

where T_t_ is the trend, S_t_ is the seasonal component, and I_t_ is the noise or random part.

In these conditions, a battery will experience changes associated with a dynamic in the form of random seasonal time series, that is, the temperature of the banks batteries will oscillate in successive diurnal warm-ups and nocturnal cooling, with higher average values in summer and lower in winter.

The damage caused to a battery due to exposure to high temperatures is not reversible, even if subjected to a subsequent cooling. For example, the corrosion effect of the positive grid cannot be eliminated and occurs at all temperatures, which is simply a matter of the speed at which the corrosion process occurs. The only solution is to try to control and avoid as far as possible the causes involved in the increase of the temperature in batteries, with the criteria of economic profitability against the risks of failure [56].

**Figure 11:**
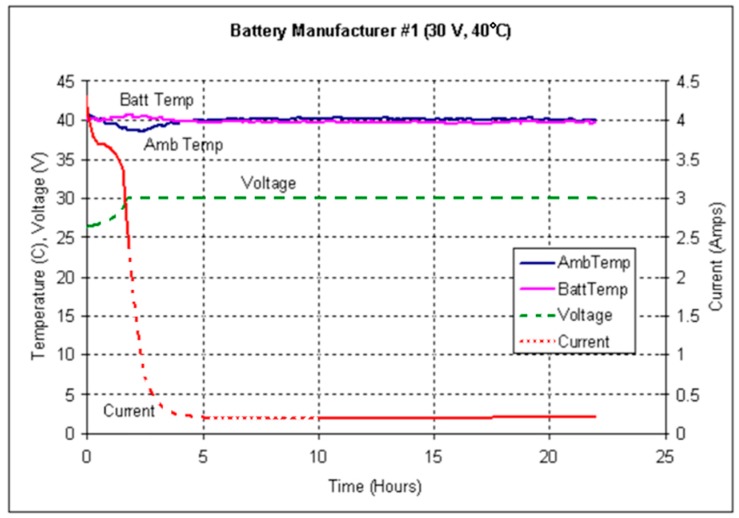
Ambient and battery temperature, voltage, and current over 23 h [61].

**Figure 12:**
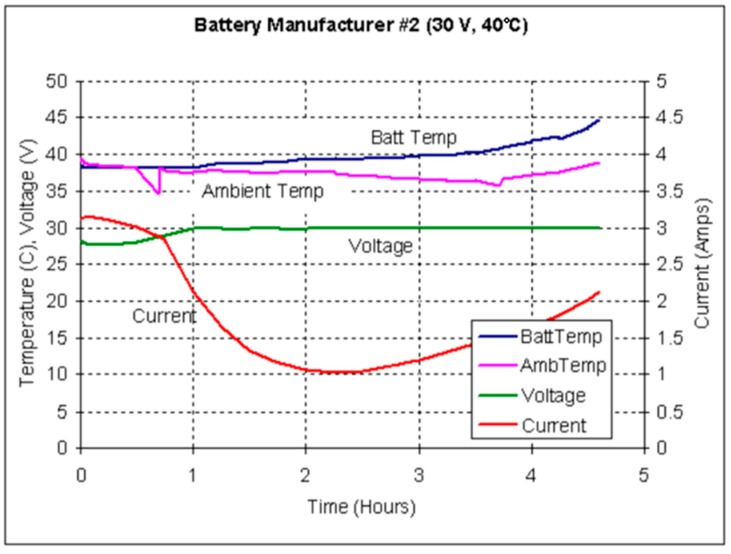
Graph showing the effects of thermal runaway on current and temperatures [61].

From a general point of view, the method developed in this work is based on the performance of a comparative analysis of the behavior of the parameters presented in Figure 11 and Figure 12, based on laboratory tests. In these tests [61], a regulatory control of the battery installation chamber temperature T_amb_ is made. Figure 11 shows a normal operating behavior of the battery, and Figure 12 shows a runaway phenomenon.

A difference introduced in the currently presented work is that the operation and tests of the batteries have been made in real navigation conditions, where the possibility of using refrigerated chambers for batteries installation is not feasible in most cases, from an economic point of view or from an energy consumption point of view.

One of the first characteristics observed in the analysis of the series recorded in the tests is the important influence that the external temperature T_ex_ has on the dynamics of the internal temperature T_in_ of the batteries, and also on the tendency of the battery installation chamber’s ambient temperature T_amb_. Both show a behavior whose dynamics are associated with random seasonal time series, influenced by the external temperature T_ex_, as can be verified by a cross-correlation analysis between the lagged series.

Note that T_in_ increase is due to the superposition of a set of parameters that overlap their effects as heat sources that increase the internal temperature of the battery. 

The first parameter to consider is the external temperature T_ex_ whose source lies in the existing weather conditions. The second parameter is the ambient temperature T_amb_, which results from the “filtering” effect caused by the installation chamber on T_ex_. The third parameter is due to the contribution to the temperature increase produced by the ripple current (AC), whose source comes from the power electronics of the battery charger specifically associated to the aging of its components (diodes). 

The float input current (I_float_) contributes to the increase in temperature and the reduction of the internal impedance of the battery, which in turn causes the battery to accept even more current, that is able to trigger a process of thermal runaway instability after a previous and accelerated dry out process [57]. The observation of a significant increase in its value in a trend analysis can be considered as a redundant indicator of the runaway failure event occurrence. 

Therefore, there are clear indicators associated with measurable parameters that redundantly predict the development of first a dry out failure, followed by a runaway process, such as the associated behavior of the T_in_ and T_amb_ temperatures on the one hand, and the I_float_ current. It should be noted that the selected parameters, T_ex_, T_amb_, T_in_, V_float,_ and I_float_ are easily accessible, taking into account that they are available in the equipment described above in the NMEA 2000 network.

Under normal operating conditions, the internal temperature T_in_ of the batteries and the temperature T_amb_ of the corresponding installation chamber will have a dynamic tracking behavior affected by a certain delay, with respect to the external environment temperature T_ex_. That is, T_in_ and T_amb_ will maintain a certain lagged correlation with T_ex_, which is justified due to the thermal inertia of the batteries and the installation chamber [56,83].

A correlation analysis between the internal temperature of the battery T_in_ and the ambient temperature T_amb_ could be made for each battery or bank of batteries in order to check if the first follows the second, or is influenced by an additional cause of failure that affects the correlation of the two series.

This work focuses on proposing a specific method for the comparative trend analysis of the internal temperature of the batteries (T_in_) and battery installation chamber temperature (T_amb_). The objective is to apply predictive fault diagnosis using early detection of the failure or loss of capacity of the battery heat dissipation process to prevent any undesirable increase in temperature, in order to avoid the development of failure modes such as dry out and thermal runaway. Taking into account that dry out is often a prior effect to other failure modes such as grid corrosion [57], this diagnosis method deals directly or indirectly with the failure modes with the highest percentage of occurrence, in addition to the most dangerous failure modes.

The advantage of the diagnostic method based on the comparative trend analysis proposed in this work is that it focuses on the assignable cause that precedes the dry out and thermal runaway failures, which is the loss of operating capacity of the heat dissipation process of the battery banks. The heat dissipation process of battery banks in real navigation conditions does not have a specific nature, but it depends on the characteristics of the installation chambers (which do not remain invariable in time), the materials used, the size of the heat dissipation areas, and the ventilation conditions, etc., and that affects the batteries in a variable way depending on the type of batteries and their intrinsic aging state condition.

If an increasing divergence through an additional comparative trend analysis of both T_in_ and T_amb_ is observed, it becomes a prior indicator that the battery is not being able to dissipate normally the heat generated inside. In other words, this first failure diagnosis of the heat dissipation process becomes a predictive indicator to avoid possible dry out and eventual thermal runaway failures.

## 6. Results and Discussion

**Figure 13:**
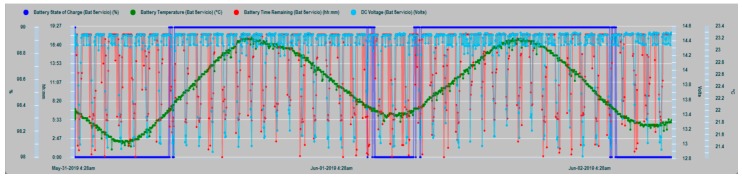
Data series of house battery bank parameters: State of Charge SOC (dark blue), Time Remaining TR (green), DC current (red), and DC Voltage (clear blue).

**Figure 14:**
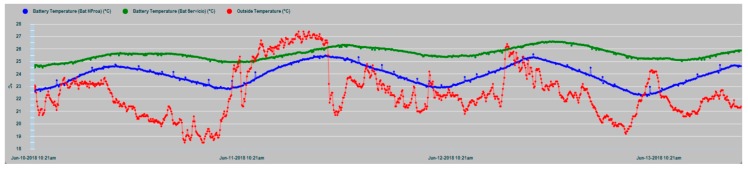
Time series of the outside temperature T_ex_ (red) and the internal temperatures T_in_ of bow thruster battery bank (blue) and house battery bank (green).

**Figure 15:**
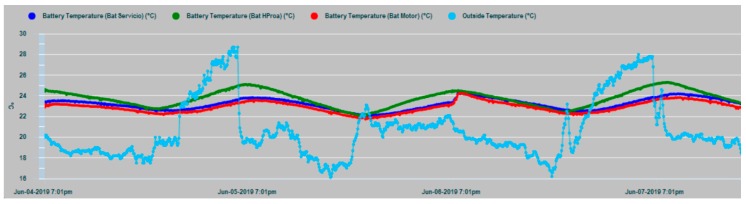
Data series of the external temperature T_ex_ (clear blue) and the three banks inner temperatures: T_in_ Bow thruster (green), T_in_ House (dark blue), and T_in_ Engine (red).

The results of the parametric measurements made and their interpretation are shown in this section. Figure 13 shows an example of the evolution of some of the battery parameters that are supervised in the N2KExtractor software display. In Figure 14 and Figure 15, the time series of the parametric values of the internal temperatures of batteries corresponding to the house battery bank, bow thruster battery bank, engine battery bank, and the external temperature T_ex_ are shown. In the dynamic behavior of the time series, it is easy to recognize their seasonal and random nature, especially in the case of the external temperature series. In both Figures, the temperatures of the battery banks are compared to the outside temperature, observing a follow-up with a certain time delay.

It should be realized that battery temperature T_in_ and external temperature T_ex_ have certain differences in their appearance, mainly due to the higher frequency components of T_ext_, while, T_in_ shape is smoothed. This is due to the large thermal inertia of the battery. It takes time for the battery to absorb temperature and it takes time for the battery to relinquish temperature. In addition, the daily cyclic behavior of all data series is completely evident.

**Figure 16:**
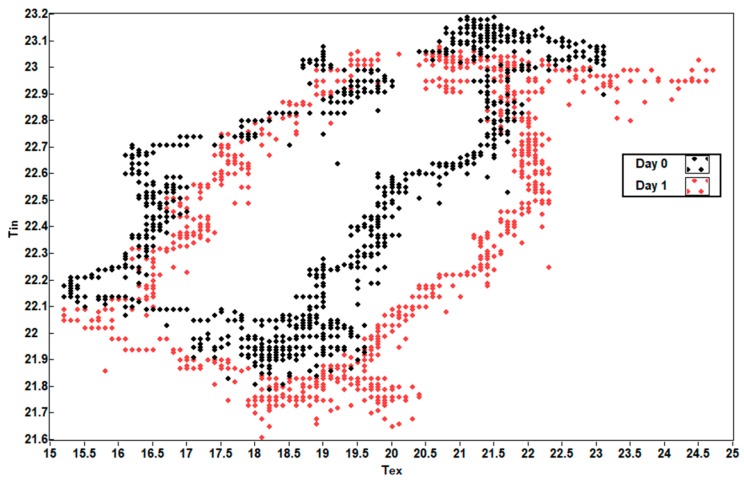
Scatter diagram of battery temperatures T_ex_ and T_in_.

Figure 16 shows that the external temperature T_ex_ and the internal temperature T_in_ of the battery are correlated with a certain delay value, as affirmation of the working hypothesis.

**Figure 17:**
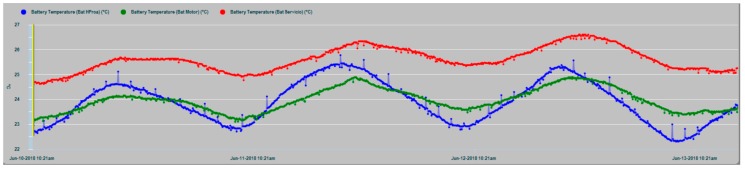
Time series of the T_in_ internal temperatures of the house battery bank (red), bow thruster battery bank (blue), and engine battery bank (green).

In Figure 17, the T_in_ temperature series of the three battery banks are shown, where maximum coincidence in the dynamic evolution of the house and engine banks exist, because in this case, they share the same installation chamber, while the bow thruster battery bank are installed in a different chamber in the bow vessel area. In all cases, it is evident that the thermal inertia and the enclosures of the installations act as smoothing filters of the battery inner temperature series, notably suppressing the high-frequency random component observed in the outdoor temperature series (T_ex_) in Figure 14 and Figure 15.

The house battery bank presents in general an increase of temperature with respect to the engine battery bank and the bow thruster battery bank, which is justified by the fact that it normally supplies a permanent power consumption to electrical and electronic equipment. Requests for energy supply from the other two banks can be described as sporadic, compared to those made to the house battery bank.

**Figure 18:**
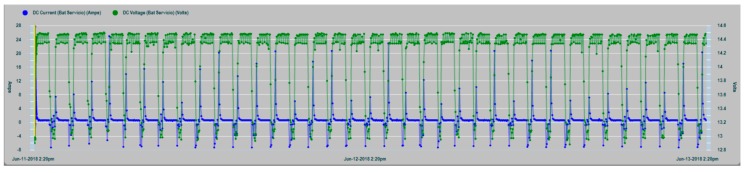
House battery bank charge cycling: DC current (blue), DC voltage (green).

In Figure 18, a graph of the charge cycles series of the house battery bank is shown, together with the values of the charge current (positive values) and discharges by consumption (negative values).

**Figure 19:**
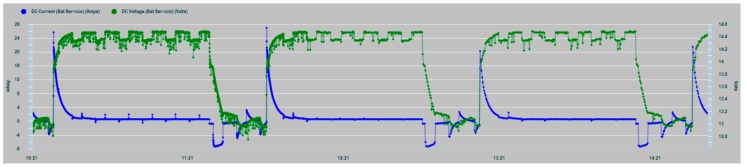
House battery bank charge cycling zoom.

In Figure 19, a zoom of the charge and consumption cycle of the house battery bank is shown with greater clarity.

**Figure 20:**
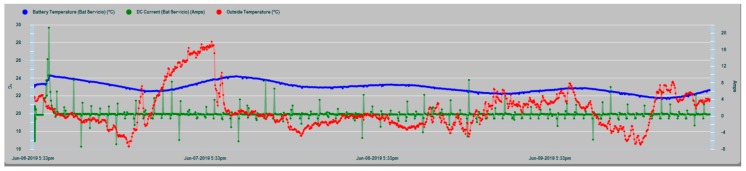
Peaks of energy demand versus temperature increases.

The peaks of energy demand (in green, Figure 20) are also clear contributors to the increase in the internal temperature of the batteries (in blue), whose response is manifested by rising ramps in the corresponding curve, especially due to the charging currents that are subsequently supplied. This justifies the convenience of monitoring the consumption and not subjecting the system to unnecessary overloads.

**Figure 21:**
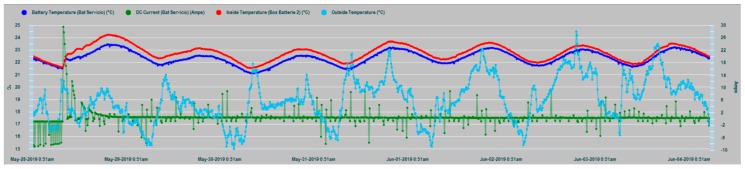
Peaks of energy demand versus temperature increases.

Figure 21 shows the effects of load demand peaks on the T_in_ and T_amb_ temperatures of the house battery. In conditions of normal operation it is observed that the curve of the installation chamber temperature T_amb_ is lifted upwards from the curve of the internal temperature T_in_ of the house battery. This is explained by the activation of the heat dissipation process from the inside of the battery to the outside in the installation chamber, which increases T_amb_. Later when the current demand stops, T_amb_ tends to overlap progressively with the internal temperature T_in_.

**Figure 22:**
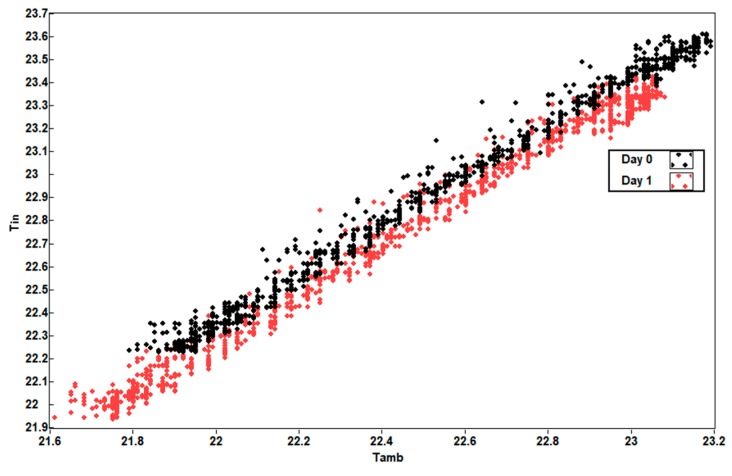
Scatter diagram of house battery temperature T_in_ and installation chamber temperature T_amb_.

Figure 22 shows the scatter diagram of battery temperatures T_in_ and T_amb,_ with a high correlation index and with a barely significant delay between both curves.

**Figure 23:**
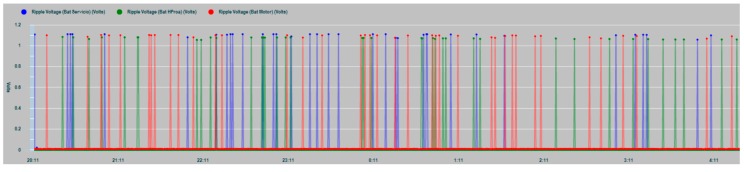
Ripple voltage data-series measurements on the three battery banks: House battery banks (dark blue), bow thruster battery banks (green), and engine battery banks (red).

In Figure 23 the ripple levels of the three battery banks are shown as a result of the measurements made in the tests. As can be seen in the graph, values exceeding 1 V are shown, which exceed the recommended referenced values of the DC voltage applied to the battery (ripple voltage smaller than half the normal float-charge conditions).

**Figure 24:**
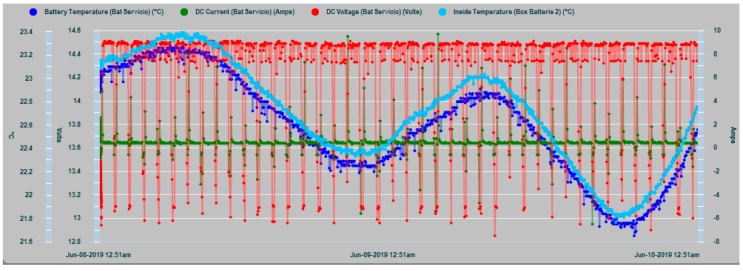
Ambient T_amb_ (clear blue), battery temperature T_in_ (dark blue), voltage V_float_ (red), and current I_float_ (green) over 48 h.

**Figure 25:**
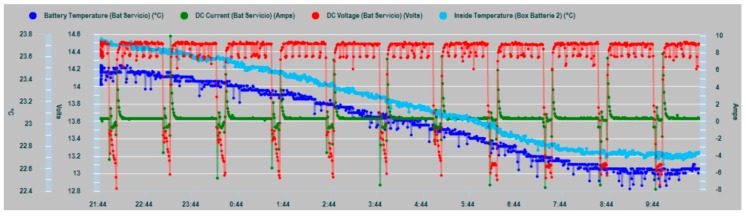
Zoom of ambient and battery temperature T_amb_ and T_in_ voltage and current over 12 h.

Figure 24 and Figure 25 shows the four parameters involved in a possible dry out and runaway phenomenon in real operating conditions based on random seasonal series dynamics. They are the internal temperature of the house battery T_in_, the temperature of the battery installation chamber T_amb_, the floating charge voltage of the battery V_float_ and the float current I_float_. Under normal operating conditions, as shown in the laboratory test of Figure 11, both temperatures should maintain a close correlation, while the float current should remain constant at the standard value of 250 mA.

In Figure 24 the convergence shown by the T_in_ and T_amb_ temperature curves is significant, whereby the heat dissipation process of the battery bank is working adequately, taking into account the specific characteristics and conditions of the type of battery and the installation chamber.

**Figure 26:**
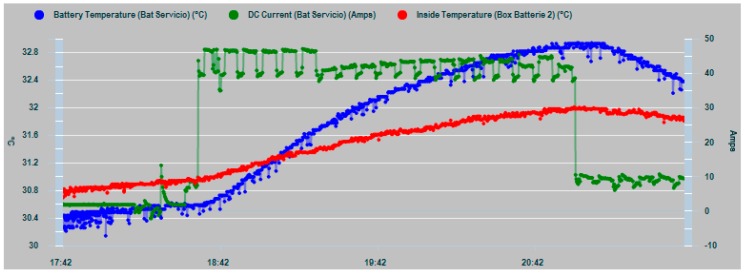
Test for divergent comparative trend analysis of T_in_ (blue) and T_amb_ (red), where the heat dissipation capacity is being exceeded prior to dry out and eventually to thermal runaway failures.

Figure 26 shows a limited partial overload test with two batteries of the vessel’s house battery bank, in order to cause a battery bank overheating. Partial overload is used for safety reasons due to the extreme proximity of the battery bank with a fuel tank. The test was carried out using a battery charger, an inverter connected to the batteries under test, and different AC loads connected to the inverter. This allowed the battery bank to be subjected to successive charges and discharges that reached values of 50 amps.

Initially Figure 26 shows a test period in which the batteries are not subjected to any special overload from 17:42 to approximately 18:30. In this period, the two temperature series involved respond normally by drawing two parallel curves with a distance between them of approximately 0.4 °C. Being the internal temperature T_in_ is lower than T_amb_, and Figure 26 shows that the dissipation process of the battery heat is working properly. When the current overload is activated, the temperatures parallel behavior is modified and a change in the ROC occurs in both curves, with a greater slope in the T_in_ curve. This results in the crossing of the curves and an increasing divergence between T_in_ and T_amb_.

In this case, the progressive divergence of the two curves shows that the heat dissipation process capacity is being exceeded and, if this condition persists, the development of the dry out phenomenon and eventually the thermal runaway occurrence is predictable.

## 7. Conclusions

For safe maritime navigation, having a monitoring system based on observing the actual condition of use and specific technical characteristics of the ship’s energy storage system is especially important and critical. The NMEA 2000 network—together with intelligent sensor devices for battery monitoring, temperature measurements, ship data logging, and associated software developed for data processing—allows for obtaining time series of data and for the application of trend analysis for better management of alarms, in order to face the appearance of incipient and/or sudden failures.

The predictive diagnostic method proposed in this study does not depend on a temperature threshold value, but on the previous failure of the heat dissipation process. This behavior demonstrates that the working hypothesis proposed in the comparative trend analysis can be used as a recognition pattern for the predictive diagnosis of eventual dry out and thermal runaway failures.

In the opinion of the authors, the equipment that is usually installed for the supervision of battery banks in navigation applications offers sufficient information on the most important parameters of battery operation. However, from the point of view of the implementation of predictive diagnostic techniques, the information they offer is not always easy to analyze by potential users who are largely not experts in the field, and much of its information potential is lost.

For truly useful decision making in critical situations, it would be desirable that the information is oriented toward the mode of failure and displayed with multi-parametric high level integration. In that way, comparative trend analyses and correlation of the involved parameters could be applied. Even more, this information should be given in the form of patterns of immediate or easy recognition, that reliably allow the detection of an assignable cause such as a failure of the heat dissipation process of the battery, useful for predictive dry out and thermal runaway fault diagnosis. It is concluded that these failure modes can be conveniently diagnosed by easy recognized patterns obtained performing comparative trend analysis to the variables measured onboard.
